# Real-time cell metabolism assessed repeatedly on the same cells *via* para-hydrogen induced polarization†

**DOI:** 10.1039/d3sc01350b

**Published:** 2023-06-20

**Authors:** Yonghong Ding, Gabriele Stevanato, Frederike von Bonin, Dieter Kube, Stefan Glöggler

**Affiliations:** a Group of NMR Signal Enhancement Max Planck Institute for Multidisciplinary Sciences Am Fassberg 11 37077 Göttingen Germany stefan.gloeggler@mpinat.mpg.de; b Center for Biostructural Imaging of Neurodegeneration University Medical Center Göttingen Von-Siebold-Str. 3A 37075 Göttingen Germany; c Clinic for Hematology and Medical Oncology University Medical Center Göttingen Robert-Koch-Str. 40 37075 Göttingen Germany

## Abstract

Signal-enhanced or hyperpolarized nuclear magnetic resonance (NMR) spectroscopy stands out as a unique tool to monitor real-time enzymatic reactions in living cells. The singlet state of para-hydrogen is thereby one source of spin order that can be converted into largely enhanced signals of *e.g.* metabolites. Here, we have investigated a parahydrogen-induced polarization (PHIP) approach as a biological assay for *in vitro* cellular metabolic characterization. Here, we demonstrate the possibility to perform consecutive measurements yielding metabolic information on the same sample. We observed a strongly reduced pyruvate-to-lactate conversion rate (flux) of a Hodgkin's lymphoma cancer cell line L1236 treated with FK866, an inhibitor of nicotinamide phosphoribosyltransferase (NAMPT) affecting the amount of NAD^+^ and thus NADH in cells. In the consecutive measurement the flux was recovered by NADH to the same amount as in the single-measurement-per-sample and provides a promising new analytical tool for continuous real-time studies combinable with bioreactors and lab-on-a-chip devices in the future.

## Abstract 2

We propose a fast protocol that enables two parahydrogen induced polarization measurements on the same cell sample and yield kinetic rates of metabolic conversion under inhibition and stimulation conditions.

## Graphical abstract

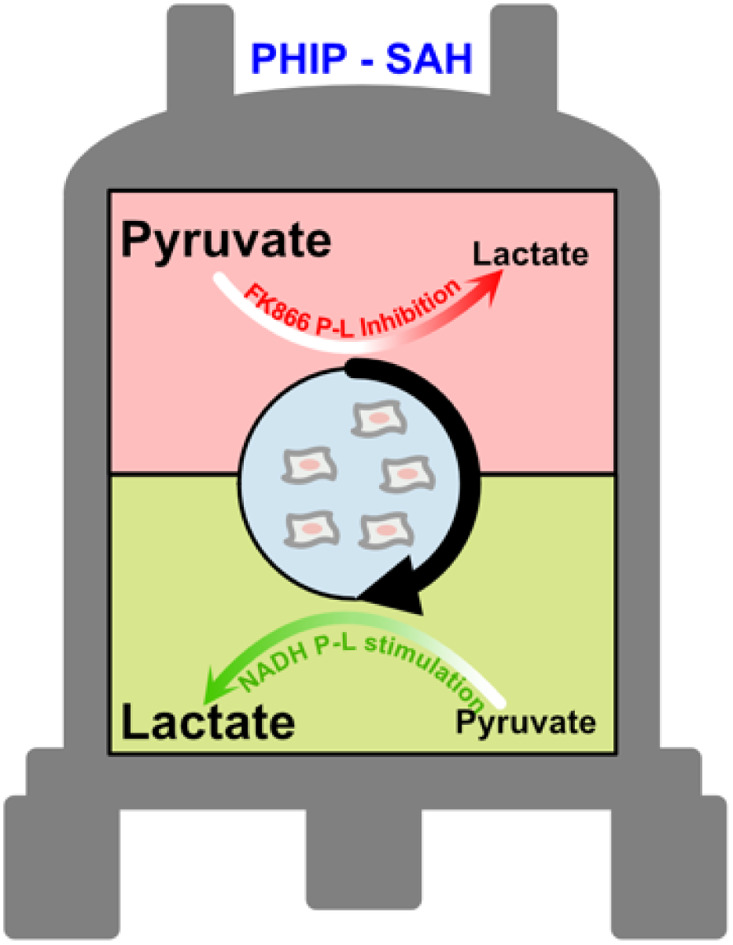

Nuclear magnetic resonance (NMR) and its variant magnetic resonance imaging (MRI) represent primary tools to probe cell metabolism both *in vitro* and *in vivo*.^1^ Among the techniques being used in metabolomics, NMR has the unique strengths of being (1) non-destructive and (2) capable of detecting signals even in deep tissue regions *in vivo*.^2^ These features allow for preclinical and clinical investigations of cell metabolism associated with diseases.^3^

The applications of NMR, however, has been largely limited by the inherently low signal sensitivity, with only about 2 over 10^5^ spins contributing to the ^1^H signal formation at 298 K and 7 T. Over the last decades, a number of hyperpolarization techniques have been developed to boost the NMR signals^4^ with dissolution dynamic nuclear polarization (dDNP) being the game changer.^5^ dDNP has been used to enhance several thousand-fold the NMR signal from biologically relevant probes used for *in vivo* monitoring of enzyme-driven metabolic reactions in preclinical and also clinical studies,^1*c*,6^ something not obtained by other analytical techniques, such as mass spectrometry,^7^ [^18^F] fluorodeoxyglucose positron emission tomography or photo-chemical assays. However, dDNP requires long experimental time (0.5–3 hours per sample) and high technical expertise.

An alternative hyperpolarization approach is para-hydrogen induced polarization (PHIP). Discovered by Bowers and Weitekamp^8^ in 1986, PHIP originates from the spin-zero hydrogen fraction named para-hydrogen (pH_2_), that is enriched by cooling hydrogen gas in the presence of a catalyst.^9^ Despite magnetically inactive, the high spin order of para-hydrogen can be revealed by NMR when the pH_2_ symmetry is broken by a hydrogenation reaction. In the Side Arm Hydrogenation variant (PHIP-SAH),^9,10^ a compound of interest is first derivatized with a structural moiety (the side arm) that contains an unsaturated group to form a precursor (Fig. 1a). Followed by the reaction with pH_2_, spin polarization transfer occurs *via* pulsed methods^11^ or magnetic field cycling^12^ from the hyperpolarized pH_2_ protons to the target ^13^C spin. Hydrolysis *via* NaOH or Na_2_CO_3_ injection cleaves off the side arm to retain the signal-enhanced ^13^C labelled metabolite of interest (Fig. 1b). Applications of PHIP-SAH to biological systems require further purification steps: removal of the organic solvent and catalyst, and pH adjustment for a biocompatible final solution (steps 1–4 in Fig. 2). Although all of these steps partially deplete the initial signal intensity, nonetheless [1-^13^C]pyruvate has been hyperpolarized by PHIP-SAH and used to probe in real-time the metabolism in-cell^13^ and *in vivo*^11*e*,14^ with the first tumor imaging recently obtained on a mouse model at 7 T.^11*e*^ We have also demonstrated carbon polarization levels of 59.7% for ethyl pyruvate, corresponding to ∼10^5^ signal enhancement at 7 T, in only few seconds of experimental time.^13*a*^ The particular advantage that we see in using para-hydrogen is that potentially high throughput experiments can be performed on many different amounts of samples and even repeatedly on the same cell type. Here we show that the fast delivery of PHIP hyperpolarization allows for multiple hyperpolarization experiments on the same cell sample, in only a few minutes with polarization levels enabling real-time metabolic studies with [1-^13^C]pyruvate.

**Fig. 1:**
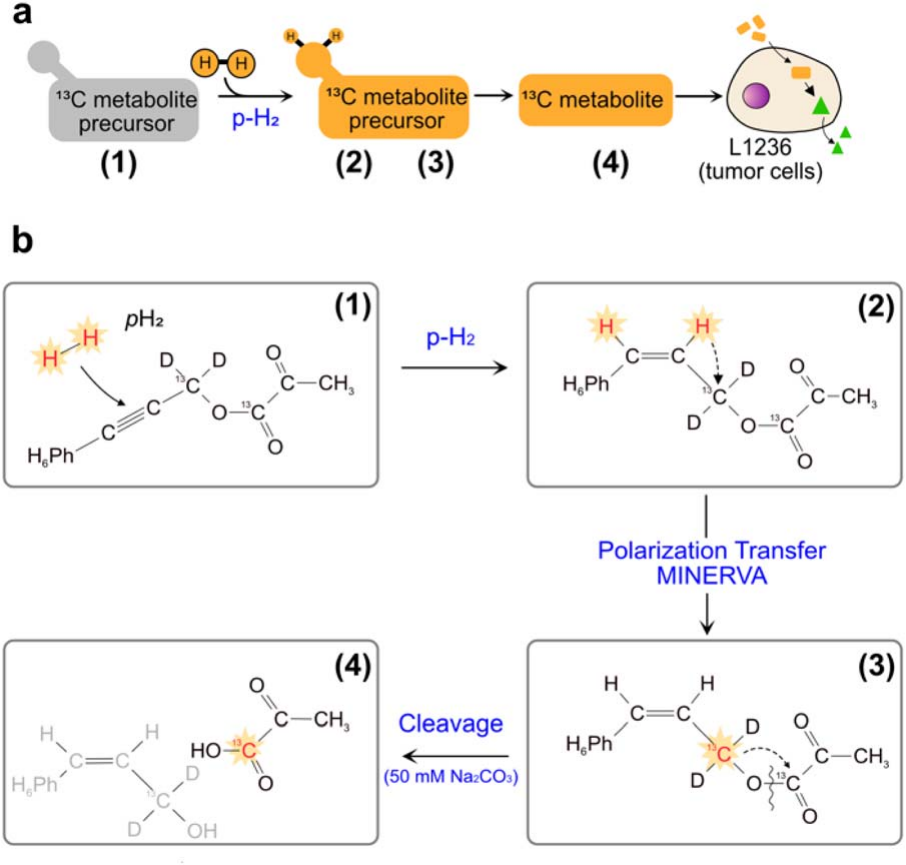
(a) Sketch of side-arm approach of PHIP for the *in vitro* cell application. Grey and orange shapes indicate non-hyperpolarized and hyperpolarized compounds, respectively. Green triangle indicates the formation of signal-enhanced products in cell *via* enzymatic reactions. The numbers (1) to (4) refer to the specific steps in making the hyperpolarized metabolite. (b) Structures of phenyl acetylene precursor used in this work (1) and its followed chemical conversions in each step of a PHIP experiment with demonstration of hyperpolarization transfer. Specifically, upon hydrogenation of the precursor with pH_2_, the high spin polarization, indicated in red and star, of protons can be transferred first to an intermediate ^13^C (2), and then to the target position (3) by applying the pulse sequence MINERVA,^11*h*^ followed by the cleavage of the precursor, by injection of 50 mM Na_2_CO_3_ in D_2_O, into hyperpolarized 1-^13^C pyruvate (4).

**Fig. 2:**
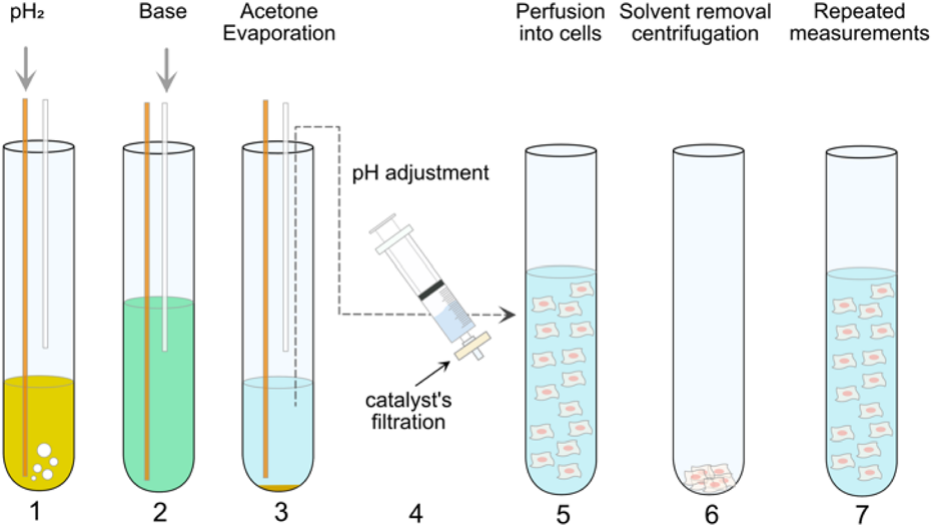
PHIP assay protocol employed in the present work. In step 1, pH_2_ is supplied through a yellow fiber into a solution of pyruvate precursor (55 mM) and catalyst (10 mM [1,4-bis(diphenylphosphino)butane](1,5-cyclooctadiene)rhodium(i) tetrafluoroborate) in *d*_6_-acetone for 20 s. Immediately after the bubbling, the high spin order of pH_2_ can be transferred from the side-arm to a ^13^C atom on the pyruvate moiety by applying MINERVA^13*a*^ sequence at 7 T. Step 2 comes the injection of 50 mM Na_2_CO_3_ solution in D_2_O *via* a plastic cannula to cleave off the side arm from the precursors. Step 3 describes the evaporation of the organic solvents *via* the outlet of the system connected to an external vacuum pump. In step 4, the solution containing hyperpolarized pyruvate was subjected to pH adjustment and filtration. In step 5, the precursor was injected into a NMR tube with cells suspended in normal medium. Step 1 to 5 are conventional protocol used for *in vitro* cell application.^13*a*^ Step 6 and 7 can be required in a Double PHIP Experiments per Sample (DES) protocol (see also Fig. 3). Step 6 and 7 describe the centrifugation of suspended cells directly in the NMR tube and the resuspension of the cells for next PHIP experiment.

A hallmark of cancer is a profound reprogramming of the cell metabolism.^15^ Glycolysis and oxidative phosphorylation (OxPhos) are simultaneously active in many cancer cells. The now called “Warburg effect” was the first description of a metabolic phenotype in tumors whereby the cells are metabolizing glucose anaerobically.^16^ This phenomenon has been widely used in hyperpolarized magnetic resonance for cancer diagnosis.^17^ As the terminal product of glycolysis, pyruvate can readily diffuse into cancer cells and convert into lactate *via* Lactate dehydrogenase (LDH), alanine *via* alanine transaminase (ALT) or to metabolites involved in the tricarboxylic acid (TCA) cycle in mitochondria. The carbonyl carbon of [1-^13^C]pyruvate is polarized by PHIP-SAH and purified using our Maximizing Insensitive Nuclei Enhancement Reached *Via* para-hydrogen Amplification (MINERVA) protocol. With a carboxyl-^13^C *T*_1_ ∼ 50 s, the signal of hyperpolarized pyruvate and its conversion product lactate can be monitored in real time up to 3 minutes by the consecutive application of small (20°) flip angle pulses. The pyruvate to lactate (P–L) conversion rate, *k*_PL_, can be extracted by established models^18^ and used to compute the flux defined as (*k*_PL_ × [Pyr(*t*_0_)])/ncells, with [Pyr(*t*_0_)] the pyruvate concentration at injection and ncells the number of cells. The P–L flux reflects the real-time level of anaerobic glycolysis in cell or *in vivo* and can be used as a biomarker to assist disease diagnostics and treatment effectiveness of different cancers.^19^ Therefore, a fast and high throughput assay to obtain real-time metabolic information upon the treatment effectiveness is desirable and helpful, especially for preclinical studies. For animal models, the amount of hyperpolarized metabolite to be administered is restricted to the animal body weight and thus it is difficult to perform multiple measurements on the same sample. However, for cellular models, the *in vitro* cells can be, in principle, fast recycled or collected and subjected to more than one measurement, saving much of cell culture efforts, the preparation time and cost. A protocol for a new procedure is suggested here that allows fast recycling of the cells from the first PHIP experiment and performing a second measurement on same sample within 15 min and still maintaining high cell viability (>90%)). When comparing findings from the initial first PHIP measurements and the subsequent second PHIP measurements on the same sample, we obtained comparable P–L fluxes from standardized Hela Kyoto wild-type cells (abbreviated as Hela cells in the following text). We used the novel methodology in a follow-up experiment to examine a lymphoma cell line. Lymphoma is a cancer originated from lymphocytes, mostly B cells. There are two main types of lymphoma:^20^ Hodgkin Lymphoma (HL)^21^ and Non-Hodgkin Lymphoma (NHL).^22^ HL has a particular appearance under the microscope, where the mononuclear Hodgkin cells and the multinucleated Reed-/Sternberg cells are comprising only 1% of the tumor mass. Cell lines from lymphoma HL are characterized by an aberrant activity of the proto-oncogene MYC, which is a main regulator of the LDHA.^23^ LDHA is a variant of Lactate dehydrogenase that catalyzes the P–L conversion. Monitoring the P–L conversion can be relevant for characterizing different subtypes of lymphoma cancer cells. Previous publications have reported the global gene expression (GE) analysis and also proteomics and metabolomics analysis of lymphoma and the lymphoma microenvironment.^24^

Here, we measure the P–L fluxes of one HL cell line, L1236, under the impact of an inhibitor of nicotinamide phosphoribosyltransferase (NAMPT), FK866. Furthermore, we conducted rescue-experiments on the same cells by supplying the FK866-treated cells with external NADH. The FK866 treatment of L1236 cells is associated with a strong reduction in NAD^+^ and thus also NADH. We found that the inhibitor can reduce the flux for L1236, but the flux could be recovered upon NADH rescue. These results were first obtained by the conventional protocol of one measurement per independent sample and were subsequently confirmed by the proposed procedure of two consecutive measurements on the same cell sample.

Previously we reported the application of PHIP assay on HEK cell models associated with Parkinson diseases.^13*a*^ The protocol employed is shown in Fig. 2 from Step 1 to 5. In this work, the same protocol (Step 1 to 5) is used but the precursor (phenyl(1-^13^C,^2^H_2_)prop-2-yn-1-yl(1-^13^C)pyruvate) is fully protonated in the phenyl ring (Fig. 1b). The deuteration of the ^13^CD_2_ part bridging pyruvate with the side arm in the precursor molecule is instrumental to the MINERVA-induced polarization transfer.^11*h*^ Avoiding the phenyl ring deuteration results in an overall simpler chemical synthesis, but it also affects the strength of the carbon hyperpolarized signal (*i.e.* the polarization level). The ^13^C polarization level of 14.3 ± 2.1% after hydrogenation for the cinnamyl precursor used here is in line with our previous report.^11*a*^ The degree of carbon polarization is in general affected by a variety of factors. Recently we showed that the carbonyl carbon in ethylpyruvate can be polarized up to ∼60%^13*a*^ in acetone-*d*_6_ (which corresponds to a signal enhancement of ∼>10^5^) using a fully deuterated precursor molecule. Maintaining the ethylpyruvate polarization level in the present study is unrealistic due to (1) a different side arm (phenyl(1-^13^C,^2^H_2_)prop-2-yn-1-yl(1-^13^C)pyruvate) *vs.* [1-^13^C]vinyl pyruvate) and (2) to the protonated phenyl ring in the cinnamyl precursor, which dilutes the hyperpolarized signal in a larger proton spin system (Fig. 1a). Instead, the proton–proton dipolar contacts serve as a more effective relaxation mechanism. Additionally, the ratio between precursor molecule and Rh catalyst used in this work is 5.5 (55 mM pyruvate precursor and 10 mM Rh catalyst). In the following, the protocol in Fig. 2 from Step 1 to 5, is referred to as single-measurement-per-sample (SES).

The cell slurry in the NMR tube can be pelleted using a bench-top hand centrifuge after one PHIP experiment, and the supernatant is removed using a plastic cannula. We then re-suspend the cells for a subsequent NMR experiment within 15 minutes using standard cell culture DMEM medium (or with NADH added) (Fig. 2 from step 6–7 and again from step 1–5).

Double experiments per sample (DES) is the term used to describe this second methodology. The time required for each step is reported in the ESI.† It is actually possible to prepare samples more quickly by doing many measurements on the same cell sample. We initially test the P–L conversion on the standardized Hela cells using 1-^13^C pyruvate to demonstrate that DES can produce relevant data. From Fig. 3a and b, the integral areas of pyruvate and lactate are extracted and the model detailed in the ESI† returned the kPL value. In the model, we assumed the pyruvate concentration perfused injected into the cells to be 4.8 mM (an average of our previous experiments, *n* > 80) for all the DES assays. For the SES assays, the concentration of the pyruvate can be obtained after PHIP experiment by a long-lasting NMR ^13^C thermal experiment, which is not possible for the DES assays. However, we obtained similar results (no statistical significant difference on applying the *t*-test model) between first and second DES experiments on the same Hela cell sample (Fig. 3c and d, *n* = 3 replicates). After the first and the second PHIP experiments, the Hela cell viability was >95%, which indicates that a repeated PHIP measurement was not harmful to the cells.

**Fig. 3:**
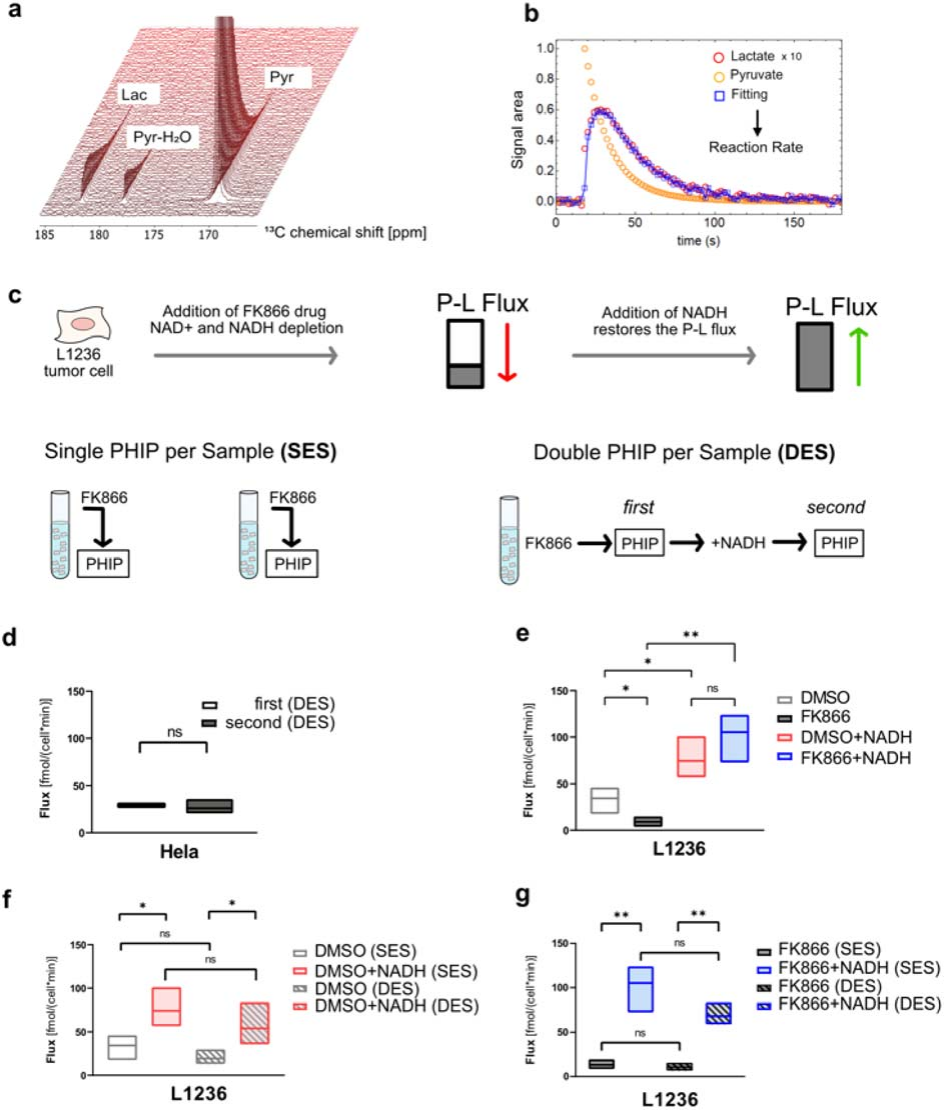
(a) Series of NMR spectra recorded over time showing the formation of lactate from pyruvate in Hela cells. (b) Time-dependent integrals of the signals can be analyzed to obtain the pyruvate-to-lactate (P–L) conversion rate. (c) Schematic protocol of SES PHIP (left) and DES PHIP (right). (d) DES on the Hela cells. The calculated fluxes of the first PHIP measurements were compared with the second PHIP measurements (*n* = 3, biological replicates). (e) SES assays on L1236 cells under different treatment conditions: DMSO, 15 nM FK866, DMSO+50 mM NADH, 15 nM FK866 + 50 mM NADH (each condition *n* = 3, biological replicates). All of these experiments were performed as SES. The treatment time of the cells of FK866 was around 25 hour. (f and g) Comparison between SES and DES. In f, DES (double) *vs.* SES (single) on L1236 cells under different treatment conditions: DMSO, DMSO+50 mM NADH. In (g), DES (double) *vs.* SES (single) on L1236 cells under 15 nM FK866, 15 nM FK866 + 50 mM NADH (each condition *n* = 3, biological replicates). The supply of NADH was 10 min before the PHIP assay. Statistical evaluation was performed using one-tail Student's *T*-test. *, *P* < 0.05; **, *P* < 0.01, ns, not significant.

Next the SES assay was performed on a HL cell line, L1236 to obtain the P–L fluxes under the impact of FK866, NAMPT inhibitor.^25^Fig. 3e shows a reduction of fluxes for L1236 after 24 hour of FK866 treatment (*n* = 3 replicates). Since the enzyme NAMPT can convert nicotinamide (NAM) into nicotinamide mononucleotide (NMN) which is the precursor of oxidized Nicotinamide adenine dinucleotide (NAD+), the malfunction of NAMPT can lead to the depletion of NAD+ and NADH in cells. NADH is necessary for the P–L conversion. Thus, the reduced availability of NADH in cell could impede the P–L conversion leading to a smaller flux. Given that the reduced level of NADH can be the cause of the reduced flux, we supplied 50 mM external NADH after the 24 hour of FK866 treatment and performed the PHIP experiment. The results (Fig. 3e) showed the flux of L1236 cells was restored and even higher compared to that of FK866 mediated reduction of NAD+/NADH and even untreated L1236 cells (*n* = 3 replicates). Next, the corresponding DES analysis was performed (Fig. 3f/g). Using the DES assay the reduction of fluxes under the impact of FK866 and more importantly also the recovery effect after the external NADH supply for L1236 cell line can be detected in a similar manner as by SES. The cell viability of L1236 was still >90% after the first and the second PHIP experiments in the DES assays. It should be noted that media and buffers were replenished quickly before the experiment to exclude effects from LDH that may be found in the extracellular environment. Thus, the DES assays have yielded similar results (no statistical significant difference) as the conventional SES assay and therefore the DES proves to be a robust method to study cellular systems especially in consecutive experiments without disturbing the cells integrity. Although our data here is presented in form of manually exchanging media we would like to note that a future application in bio-reactors is in particular appealing. It promises to be less stressful for the cells, although our results suggest minimal cell stress already for the investigated cell lines, and could allow for measuring cell responses in an even faster manner as cell media is faster restored.

For the first time, the PHIP-based methodology was applied as biological assay to test for therapeutic efficacy and recovery studies on *in vitro* cell samples. The information of real-time cell metabolism can be valuable for early disease diagnosis and treatment-response assessment. However, this is still a big challenge for other analytical techniques both *in vitro* and *in vivo*. Thus, further development of this assay is currently on the way and could facilitate a much better understanding of general cancer biology, paving the way to develop new approaches and tools for cancer diagnosis and therapy monitoring.

The DES assay could actually speed up the above therapeutic efficacy and recovery studies by measuring the FK866- or DMSO-treated cells in the first PHIP measurement (Fig. 2, step 1 to 5) and then measuring the NADH-rescued cells in the second PHIP measurement (Fig. 2, step 6–7 and then back to step 1–5). By doing so, the replicates of the cell samples needed for the experiments can be reduced by half, saving much of the efforts and time especially for experiments with large amounts of samples.

An even larger number of experiments on a single cell sample might be performed each hour by automation of the method and in combination with bio-reactors. This may even become more interesting when the suggested methodology is used in conjunction with microfabricated detecting coils.^26^ While maintaining a high number of experiments each hour, the cell number might be decreased further by around 3 orders of magnitude (to 10^4^ from 10^7^ cells). We expect our work can also find applications in studies interested in time effects on metabolic reaction rates of the same cell sample.

## Conclusions

In conclusion, our work has exploited the potentials of PHIP-based methodology to yield real-time cell metabolism information for therapeutic efficacy and drug recovery research by using a novel and high throughput DES assay. We established the validity of the protocol on a standardized HeLa cell line and assessed the impact of anti-cancer therapeutic FK866 on the metabolism of lymphoma cell L1236 using the P–L conversion rates as a biomarker. The impact of the inhibitor can be recovered by externally supplying the cells with the missing building block NADH. The novel biological assay can help us better understand the mechanisms underlying cancer and treatment resistance, as well as add to the variety of analytical approaches available for examining cell metabolism. Especially, we envision that micro-fabricated setups in combination with continuously operated bioreactors can immensely speed up analytics processes even beyond the proof-of-principle studies performed here.

## Data availability

Data is available in the ESI† or upon request from the authors.

## Author contributions

S. G. and D. K. conceived the project. Y. D., G. S., F. v. B. performed the experimental studies. S. G. and D. K. supervised the research. G. S. wrote the original draft of the manuscript which was edited by all authors. Y. D. and G. S. contributed equally to this work.

## Conflicts of interest

There are no conflicts to declare.

## Supplementary Material

SC-014-D3SC01350B-s001

## References

[cit1] Emwas A. H., Roy R., McKay R. T., Tenori L., Saccenti E., Gowda G. A. N., Raftery D., Alahmari F., Jaremko L., Jaremko M., Wishart D. S. (2019). Metabolites.

[cit2] Symms M., Jäger H. R., Schmierer K., Yousry T. A. (2004). J. Neurol., Neurosurg. Psychiatry.

[cit3] Fuss T. L., Cheng L. L. (2016). Top. Magn. Reson. Imaging.

[cit4] Kovtunov K. V., Pokochueva E. V., Salnikov O. G., Cousin S. F., Kurzbach D., Vuichoud B., Jannin S., Chekmenev E. Y., Goodson B. M., Barskiy D. A., Koptyug I. V. (2018). Chem. – Asian J..

[cit5] Ardenkjær-Larsen J. H., Fridlund B., Gram A., Hansson G., Hansson L., Lerche M. H., Servin R., Thaning M., Golman K. (2003). Proc. Natl. Acad. Sci. U. S. A..

[cit6] Kurhanewicz J., Vigneron D. B., Ardenkjaer-Larsen J. H., Bankson J. A., Brindle K., Cunningham C. H., Gallagher F. A., Keshari K. R., Kjaer A., Laustsen C., Mankoff D. A., Merritt M. E., Nelson S. J., Pauly J. M., Lee P., Ronen S., Tyler D. J., Rajan S. S., Spielman D. M., Wald L., Zhang X., Malloy C. R., Rizi R. (2019). Neoplasia.

[cit7] Jacob M., Lopata A. L., Dasouki M., Abdel Rahman A. M. (2019). Mass Spectrom. Rev..

[cit8] Bowers C. R., Weitekamp D. P. (1986). Phys. Rev. Lett..

[cit9] Hovener J. B., Pravdivtsev A. N., Kidd B., Bowers C. R., Gloggler S., Kovtunov K. V., Plaumann M., Katz-Brull R., Buckenmaier K., Jerschow A., Reineri F., Theis T., Shchepin R. V., Wagner S., Bhattacharya P., Zacharias N. M., Chekmenev E. Y. (2018). Angew. Chem., Int. Ed. Engl..

[cit10] Reineri F., Boi T., Aime S. (2015). Nat. Commun..

[cit11] Korchak S., Yang S., Mamone S., Glöggler S. (2018). ChemistryOpen.

[cit12] Reineri F., Boi T., Aime S. (2015). Nat. Commun..

[cit13] (a) Ding Y. , Korchak S., Mamone S., Jagtap A. P., Stevanato G., Sternkopf S., Moll D., Schroeder H., Becker S., Fischer A., Gerhardt E., Outeiro T. F., Opazo F., Griesinger C., Glöggler S., Chem.: Methods, 2022, e202200023

[cit14] Cavallari E., Carrera C., Sorge M., Bonne G., Muchir A., Aime S., Reineri F. (2018). Sci.
Rep.

[cit15] Hanahan D. (2022). Cancer Discovery.

[cit16] Warburg O. (1956). Science.

[cit17] Fiedorowicz M., Wieteska M., Rylewicz K., Kossowski B., Piątkowska-Janko E., Czarnecka A. M., Toczylowska B., Bogorodzki P. (2021). Biocybern. Biomed. Eng..

[cit18] Khegai O., Schulte R. F., Janich M. A., Menzel M. I., Farrell E., Otto A. M., Ardenkjaer-Larsen J. H., Glaser S. J., Haase A., Schwaiger M., Wiesinger F. (2014). NMR Biomed..

[cit19] Boudreau A., Purkey H. E., Hitz A., Robarge K., Peterson D., Labadie S., Kwong M., Hong R., Gao M., Del Nagro C., Pusapati R., Ma S., Salphati L., Pang J., Zhou A., Lai T., Li Y., Chen Z., Wei B., Yen I., Sideris S., McCleland M., Firestein R., Corson L., Vanderbilt A., Williams S., Daemen A., Belvin M., Eigenbrot C., Jackson P. K., Malek S., Hatzivassiliou G., Sampath D., Evangelista M., O'Brien T. (2016). Nat. Chem. Biol..

[cit20] (c) Swerdlow S. H. , Campo E., Harris N. L., Jaffe E. S. and Pileri S. A., WHO Classification of Tumours of Haematopoietic and Lymphoid Tissues, International agency for research on cancer, 2008, vol. 2

[cit21] Momotow J., Borchmann S., Eichenauer D. A., Engert A., Sasse S. (2021). J. Clin. Med..

[cit22] Singh R., Shaik S., Negi B. S., Rajguru J. P., Patil P. B., Parihar A. S., Sharma U. (2020). J Family Med. Prim. Care.

[cit23] Shim H., Dolde C., Lewis B. C., Wu C. S., Dang G., Jungmann R. A., Dalla-Favera R., Dang C. V. (1997). Proc. Natl. Acad. Sci. U. S. A..

[cit24] Feist M., Kemper J., Taruttis F., Rehberg T., Engelmann J. C., Gronwald W., Hummel M., Spang R., Kube D. (2017). Int. J. Cancer.

[cit25] Hasmann M., Schemainda I. (2003). Cancer Res..

[cit26] Eills J., Hale W., Utz M. (2022). Prog. Nucl. Magn. Reson. Spectrosc..

