# Supplementary material for: Real-time cell metabolism assessed repeatedly on the same cells via para-hydrogen induced polarization
Source: Chem Sci. 2023 Jun 20;14(28):7642–7. doi: 10.1039/d3sc01350b (PMC10355108; doi:10.1039/d3sc01350b)
Supplement: SC-014-D3SC01350B-s001 [file SC-014-D3SC01350B-s001.pdf]

Electronic Supplementary Material (ESI) for Chemical Science.
This journal is © The Royal Society of Chemistry 2023

Supporting Information

Real-time cell metabolism assessed repeatedly on the same cells via para-hydrogen induced polarization

Yonghong Ding†[a,b], Gabriele Stevanato†[a,b], Frederike von-Bonin[c], Dieter Kube[c], Stefan Glöggler*[a,b]

1. Cell preparation

Hodgkin lymphoma derived L1236 was kindly provided by v. Diehl (Cologne/Germany) (Peripheral blood mononuclear cells of a patient with advanced Hodgkin's lymphoma give rise to permanently growing Hodgkin-Reed Sternberg cells)[1,2]. L1236 cells were cultured in RPMI1640 (Anprotec AC-LM-0058) supplemented with 10% Fetal calf serum (Sigma) and Glutamine (Thermo-Fisher scientific). L1236 were suspension cells grown at a cell density of $5x10e^5$ cells/mL. Around 25 hrs before PHIP measurements cells were treated with 15 nM FK866 (Sigma) or corresponding amounts of DMSO (Ctrl).

Around 20-30 million cells were collected into a falcon tube and pelleted by centrifugation under 130 × g for 3 min at room temperature. The supernatant was removed and the cells were resuspended with 120 µL fresh RPMI1640 medium. The cell slurry was then transferred into a 5 mm NMR tube via a cannula for PHIP experiments. Cell viability was performed before and after PHIP experiments via using tryphan blue staining.

Hela Kyoto wild-type cell (Hela cell) was kindly provided by Dr. Peter Lenart (Göttingen/Germany). Hela cells were adherent cells cultured in the Dulbecco's Modified Eagle Medium supplemented with 4.5 g/L D-Glucose, 2 mM glutamine, 1 mM Sodium Pyruvate, 10% (v/v) heat-inactivated fetal bovine serum, 100 U/mL Penicillin and Streptomycin (Thermo-Fisher scientific). The cells were kept at 37°C in a humidified 5% CO_2 atmosphere to around 70-80% confluency before collection[3]. Upon collection, the Hela cells were detached from cell culture flask by using trypsin/EDTA (0.05%/0.02%) and collected the same as for L1236 cells.

2. NMR experiments

For all of our studies we used a phenyl acetylene [1-^{13}C] pyruvate which is synthesized as described in a previous publication[4] and used as a PHIP–SAH precursor molecule. Experimentally, 150 µL acetone-*d*$_6$ solution containing 55 mM precursor and 10 mM of [1,4-Bis(diphenylphosphino)butane](1,5-cyclooctadiene)rhodium(I) tetrafluoroborate catalyst is degassed by bubbling N_2 at ambient temperature and pressure for ~60 seconds. The experiment proceeds according to the following steps (The time required to finish each step is added at the end):

1) The 5 mm NMR tube containing the sample is immersed in a water bath for 90-120 seconds at ~60 °C for a better catalyst activation (t=0s).
2) The NMR tube is placed inside the 300 MHz spectrometer in which the probe temperature is at 37 °C (t=~5s).
3) Parahydrogen at 7 bars is supplied inside the NMR tube through a capillary (O.D ~0.1 mm) for 20 seconds. The MINERVA sequence transfers polarization from the parahydrogen protons to carbonyl ^{13}C carbon of the precursor molecule according to the spin dynamics model already described[4-7]. The parameters used for MINERVA assumed the following J-coupling network: J_{HH}=11.60 Hz, J_{H1C1}=0 Hz, J_{H2C1}=11.60 Hz, J_{C1C2}=2.68 Hz (t=~35s).
4) In the following 5 seconds, the pressure is released and 150 µL of a 50 mM solution of Na_2CO_3 in D_2O are injected into the NMR tube via a plastic cannula (i.d. 1 mm) coupled externally to a 1 mL syringe. Upon injection of the aqueous solution, the drop in catalyst's solubility initiates the catalyst precipitation (t=~40s).
5) Following the base injection, the sample is manually transferred to the top of the 7T magnet and immersed in a falcon tube containing a warm bath of ~60 °C anchored to a magnetic plate providing a residual magnetic field of ~ 8 mT (t=~45s).
6) A vacuum pump connected to the NMR tube is activated for 15 seconds to evaporate the acetone (boiling point of 56 °C) from the acetone-D_2O mixture present in the NMR tube (t=~60s).
7) The NMR tube is now opened and the hyperpolarized solution is pulled out from the NMR tube through a second 1 mL syringe and mixed with 150 µL (2X) Phosphate buffered Saline solution to adjust the aqueous pH to ~7 (the right volume of PBS buffer to add to

achieve pH ~7 was determined using a pH meter and confirmed also by pH indicator papers using samples of cells after PHIP experiments) and obtain isotonicity (t=~65s) .

8) Finally, the solution is injected through a 1 mm glass fibre filter, via a 1 mm o.d. plastic cannula, into a second NMR tube – also anchored to the magnetic plate – containing ~200 μL of cell solution previously prepared. The new solution containing hyperpolarized pyruvate and the cells to be examined is gently mixed for ~3 sec and the NMR tube is dropped inside the magnet (t=~70s).
9) The carbon NMR signal is acquired via the application of consecutive 22.5 flip angle pulses every 2 seconds (t=~3min).

For the second PHIP experiments on the same sample, the following steps are performed:

10) The cell suspension after the first PHIP experiments is palleted by using a bench-top hand-centrifuge under 80 × g for 2 min at room temperature (t=~7min).
11) The supernatant is carefully removed without removing cells and fresh medium or medium containing 50 mM NADH is added to re-suspend the cells (t=~10min).
12) The new cell suspension is subjected to a second PHIP experiment following the steps from 1)-9) (t=13-15min).

For SES protocol, the sample was subjected to NMR ^{13}C thermal measurement after PHIP experiment to estimate the concentration of 1-^{13}C lactate in the cell suspension. For DES protocol, the concentration of 1-^{13}C lactate was assumed to be 0.048 mM. It takes within 15 min to finish a DES assay. The results of cell viability after the first and second PHIP experiment in a DES assay have been shown in Table 1-3.

Table 1: Cell viability of Hela cells after the first PHIP and second PHIP experiments in a DES assay (n=3).

	Hela	
n	first	second
1	98%	97%
2	97%	95%
3	97%	97%

Table 2: Cell viability of L1236 cells after the first PHIP and second PHIP experiments under the condition

of DMSO and DMSO+NADH (n=3).

n	L1236			
	DMSO		DMSO+NADH	
	First	Second	First	Second
1	96%	95%	94%	91%
2	94%	92%	95%	93%
3	97%	93%	95%	92%

Table 3: Cell viability of L1236 cells after the first PHIP and second PHIP experiments under the condition of FK866 and FK866+NADH (n=3).

n	L1236			
	FK866		FK866+NADH	
	First	Second	First	Second
1	92%	90%	93%	92%
2	94%	91%	95%	91%
3	95%	90%	95%	93%

3. Calculation of kinetic rates

The model for calculating of the conversion rate (flux, R) of pyruvate to lactate has been described previously[3].

The equation to calculate the flux *R* using the build-up rate *k* and normalizing on initial pyruvate concentration [Lac] , and number of cells (#cells):

$$R = \frac{k \cdot [Lac]}{\#cells}$$

These results were then further analysed for statistical significance using one-tailed student t-test.

As the pyruvate-to-lactate reaction is predominantly shifted towards lactate and in accordance

with previously reported studies, we assume an unidirectional conversion rate:

$$Pyr\ kPL{\rightarrow}Lac \qquad \text{(SI.1)}$$

A set of two differential equations models the variation of the pyruvate and lactate signals over time:

$$\frac{dPyr}{dt} = -k_{PL} \times Pyr - \frac{Pyr}{T_1} \qquad \text{(SI.2)}$$

$$\frac{dLac}{dt} = k_{PL} \times Pyr - \frac{Lac}{T_1} \qquad \text{(SI.3)}$$

Following the discretized version in reference [8] equations SI.2 and SI.3 become:

$$\frac{\Delta L_k}{TR} = k_{PL} \times Pyr_k - R_{eff} \times Lac_k \qquad \text{(SI.4)}$$

$$R_{eff} = \frac{1}{T_{1,Lac}} + \frac{1 - coscos\,\beta}{TR} \qquad \text{(SI.5)}$$

The index *k* represent the *k*th slice in the pseudo 2D experiment consisting in the repeated application of 13C flip angle pulses repeated every *TR*. The effective decay rate *Reff* accounts for signal decay due to Boltzmann thermalization, repetitive excitation with flip angle pulse (20 degree for *in-vitro* experiments). The pyruvate and lactate integral signal then results in a system of linear equations for k_{PL} and R_{eff} that can be solved by a pseudo matrix inversion:

$$\left(\frac{\Delta L_1}{TR} \vdots \frac{\Delta L_k}{TR}\right) = \left(Pyr_1 \vdots Pyr_k \ - Lac_1 \vdots \ - Lac_k\right) \cdot \left(k_{PL}\ R_{eff}\right) \qquad \text{(SI.6)}$$

For every slice *k* by the integral values Pyr_k and Lac_k. By pseudoinverting the matrix above k_{PL} and R_{eff} can be estimated.

References:

1 Wolf, J. *et al.* Peripheral blood mononuclear cells of a patient with advanced Hodgkin's lymphoma give rise to permanently growing Hodgkin-Reed Sternberg cells. *Blood* **87**, 3418-3428 (1996).

2 Kube, D. *et al.* STAT3 is constitutively activated in Hodgkin cell lines. *Blood* **98**, 762-770, doi:10.1182/blood.v98.3.762 (2001).

3 Ding, Y. *et al.* Rapidly Signal-enhanced Metabolites for Atomic Scale Monitoring of Living Cells with Magnetic Resonance. *Chemistry–Methods*, e202200023, doi:https://doi.org/10.1002/cmtd.202200023.

4 Korchak, S., Mamone, S. & Gloggler, S. Over 50 % (1)H and (13)C Polarization for Generating Hyperpolarized Metabolites-A para-Hydrogen Approach. *ChemistryOpen* **7**, 672-676, doi:10.1002/open.201800086 (2018).

5 Ding, Y. *et al.* Rapidly Signal-enhanced Metabolites for Atomic Scale Monitoring of Living Cells with Magnetic Resonance. *Chemistry–Methods* **n/a**, e202200023, doi:https://doi.org/10.1002/cmtd.202200023.

6 Korchak, S., Yang, S., Mamone, S. & Gloggler, S. Pulsed Magnetic Resonance to Signal-Enhance Metabolites within Seconds by utilizing para-Hydrogen. *ChemistryOpen* **7**, 344-348, doi:10.1002/open.201800024 (2018).

7 Korchak, S., Emondts, M., Mamone, S., Blumich, B. & Gloggler, S. Production of highly concentrated and hyperpolarized metabolites within seconds in high and low magnetic fields. *Phys Chem Chem Phys* **21**, 22849-22856, doi:10.1039/c9cp05227e (2019).

8 Khegai, O. *et al.* Apparent rate constant mapping using hyperpolarized [1-(13)C]pyruvate. *NMR Biomed* **27**, 1256-1265, doi:10.1002/nbm.3174 (2014).
